# Burnout Is Associated with Reduced Parasympathetic Activity and Reduced HPA Axis Responsiveness, Predominantly in Males

**DOI:** 10.1155/2015/431725

**Published:** 2015-10-18

**Authors:** Wieke de Vente, Jan G. C. van Amsterdam, Miranda Olff, Jan H. Kamphuis, Paul M. G. Emmelkamp

**Affiliations:** ^1^Department of Clinical Psychology, University of Amsterdam, Weesperplein 4, 1018 XA Amsterdam, Netherlands; ^2^Research Institute Child Development and Education, University of Amsterdam, P.O. Box 15776, 1001 NG Amsterdam, Netherlands; ^3^Amsterdam Institute for Addiction Research, Academic Medical Center, P.O. Box 75867, 1070 AW Amsterdam, Netherlands; ^4^Center for Psychological Trauma, Department of Psychiatry, Academic Medical Center, P.O. Box 22660, 1100 DD Amsterdam, Netherlands; ^5^Netherlands Institute for Advanced Study, Meijboomlaan 1, 2242 PR Wassenaar, Netherlands; ^6^The Center for Social and Humanities Research, King Abdulaziz University, P.O. Box 80 202, Jeddah 21589, Saudi Arabia

## Abstract

There is mounting evidence that burnout is a risk factor for cardiovascular disease (CVD). Stress-related dysregulation of the sympathetic and parasympathetic system and the hypothalamic pituitary adrenal (HPA) axis may explain the enhanced risk for CVD. To test this hypothesis, 55 patients (34 males and 21 females) with burnout on sickness absence and 40 healthy participants (16 males and 24 females) were exposed to a psychosocial stressor consisting of mental arithmetic and public speech. Physiological variables (i.e., blood pressure, heart rate, cardiac output, vascular resistance, cortisol, and alpha-amylase) were measured. Basal levels, reactivity, and recovery were compared between groups. In male patients, baseline systolic blood pressure was higher, whereas basal alpha-amylase and cortisol reactivity were lower than in healthy males. In female patients, a tendency for lower basal cortisol was found as compared to healthy females. Furthermore, reduced basal heart rate variability and a trend for elevated basal cardiac output were observed in both male and female patients. Burnout is characterised by dysregulation of the sympathetic and parasympathetic system and the HPA axis, which was more pronounced in males than in females. This study further supports burnout as being a risk factor for CVD through dysregulation of the sympathetic and parasympathetic system and the HPA axis.

## 1. Introduction

Burnout is a state that results from prolonged exposure to work-related stressors and goes along with health complaints (see Lindblom et al. [1], for an overview; see [2, 3]). Burnout complaints include emotional exhaustion, negative attitudes towards work, and a sense of diminished competence to fulfil the demands posed by the job [2, 3]. Burnout is accompanied by distress including affective (e.g., depressed mood), physical (e.g., fatigue), cognitive (e.g., concentration problems), and behavioural (e.g., sleeping problems) symptoms [3]. Burnout, and more generally work-related stress, is a risk factor for cardiovascular disease (CVD; see Melamed et al. [4] and Belkic et al. [5], for reviews; see [6]). In this study we aimed to identify physiological stress mechanisms that may explain this relationship by assessing indices of main physiological stress systems, that is, the sympathetic system, the parasympathetic system, and the hypothalamic pituitary adrenal (HPA) axis [7, 8], associated with a clinical level of burnout. Assessing indices of various physiological stress systems allows for an initial step in the development of an integrative view on physiological stress adaptation that may mediate the burnout-CVD association.

Physiological stress mechanisms that are hypothesised to mediate the association between stress and CVD by promoting adverse health processes associated with CVD, including the metabolic syndrome and atherosclerosis, are sustained enhanced sympathetic activity, reduced parasympathetic vagal activity, enhanced sympathetic* reactivity* and/or delayed sympathetic* recovery* after stressor exposure, and dysregulation of the HPA axis [4, 5, 9–14]. Support for an association between these physiological mechanisms in association with CVD has indeed been obtained. For example, in their review, Palatini and Julius [13] report support for sustained enhanced sympathetic activity in the development of CVD. Furthermore, evidence for a role of reduced parasympathetic vagal activity in CVD development has been found in a large population study (*N* = 14.672) [15]. In addition, two reviews [16, 17] report support for a relationship between enhanced sympathetic* reactivity* and development of CVD, including the metabolic syndrome and atherosclerosis. Finally, a body of evidence associates dysregulation of the HPA axis with CVD (see Melamed et al. [4], for a review).

However, results on sympathetic and parasympathetic activity and HPA axis activity in association with work-related stress have been mixed (e.g., see Danhof-Pont et al. [18] for a review; see [4, 19–26]). The null-findings that were found in several studies including relatively healthy samples or employees that were still able to carry out their work despite the presence of stress complaints may be partly due to the* healthy worker effect* or restriction of range. To prevent selection bias due to inclusion of solely healthy, hardy, individuals and to ensure that exposure to stressors had resulted in a serious state of distress, we chose to include a clinical burnout sample consisting of employees that had called themselves sick because of severe work-related stress complaints and compared their physiological profile with that of a healthy reference sample. Selection of a clinical sample also ensured that stressor exposure duration had been sufficient to expect physiological stress adaptation. Another source of mixed results may be inconsistent consideration of gender differences in physiological stress responses. There is evidence to suggest that cardiovascular and neuroendocrine responding while under stress differs between the sexes due to biological (e.g., hormonal) and psychological differences (e.g., appraisal and coping) [27–29]. The generally lower physiological stress-reactivity in women between puberty and menopause in comparison to men may conceal physiological adaptation in combined samples. Moreover, as gender differences have been previously demonstrated in the relation between job strain and CVD risk [5], physiological profiles may differ between males and females with burnout. We therefore chose to assess gender differences in physiological adaptation associated with burnout.

To our knowledge, our previous study [30] on physiological adaptation in burnout was the first that combined autonomic and neuroendocrine stress indices in a clinical sample. In this study, we found support for a hyperactive physiological state as evidenced by elevated heart rate in rest and during a psychosocial stressor and elevated cortisol levels at the moment of awakening in the patient group with burnout complaints. Our results regarding stress-reactivity during a psychosocial stressor were inconclusive though, and our sample size did not allow for subgroup-analyses based on gender. The current study replicates and extends our earlier work [30] by including a larger sample enabling the assessment of gender differences in stress-responsiveness. Furthermore, with the aim to further elucidate the physiological mechanisms that may explain the association between work-related stress and CVD, we included additional measures relevant for CVD-promoting processes, that is: (a) cardiac output, (b) vascular resistance, (c) heart rate variability, and (d) alpha-amylase. Cardiac output and vascular resistance reflect well the haemodynamic function and they are the main determinants of blood pressure. More importantly, cardiac output and vascular resistance enable detection of physiological adaptation related to the development of hypertension that may not be detected by measuring blood pressure alone [31]. Rapid, beat-to-beat, heart rate variability, an accepted measure of cardiac parasympathetic or vagal activation [32, 33], was added to obtain a relatively pure measure of parasympathetic activation. Alpha-amylase was added as an additional measure of change in the sympathetic tone when studied during a psychosocial stressor [34].

In sum, our aim was to examine physiological mechanisms that may mediate the association between work-related stress and CVD. Initial support for a mediating role of physiological mechanisms would be to demonstrate the presence of adverse cardiovascular and neuroendocrine profiles in patients with burnout, which we addressed in this study. We hypothesised that burnout would be associated with sympathetic predominance in the sympathetic vagal balance and dysregulation of the HPA axis. Since Kudielka et al. [21] stated that ongoing chronic stress is generally associated with a hyperactive HPA axis and our sample was recruited within weeks to a few months after having called themselves sick, we expected to find support for a hyperactive rather than a hypoactive HPA axis. To test our hypotheses, we compared a clinical sample with burnout with a healthy reference sample on indices of sympathetic activity, parasympathetic activity, and HPA axis activity. Since burnout is not included as diagnostic classification category in the DSM-IV [35] or ICD-10 [36], the criteria for undifferentiated somatoform disorder (DSM-IV) or neurasthenia (ICD-10) are commonly used, with the added criterion that the main cause of the symptoms is work-related [37]. Comparisons were made during rest situations as well as during a psychosocial stressor. For reasons of ecological validity, we chose a psychosocial stressor consisting of a task that required mental effort (mental arithmetic) and social performance (speech task). We predicted higher levels of basal heart rate, blood pressure, alpha-amylase, and cortisol and a lower heart rate variability in burnout patients compared to healthy individuals. Second, we predicted larger reactivity and less recovery of these measures in patients compared to healthy individuals. Third, since males were expected to demonstrate larger physiological stress-reactivity as indicated by cardiovascular and HPA axis indices [27–29], we predicted to find more between-group differences for males than for females.

## 2. Methods

### 2.1. Participants

Eighty-five patients were recruited through occupational physicians, general practitioners, and by self-referral to participate in a RCT about treatment efficacy of stress-management training (e.g., de Vente et al. [38]). Twenty-seven patients did not fulfil the inclusion criteria (e.g., having major depression as a primary diagnosis) or refused to take part in the study. Of the remaining 58 patients, 43 were recruited through occupational physicians, 3 through general practitioners, and 12 by self-referral to our study. Eligibility was based on a telephone screening interview by a clinical psychologist that assessed the presence of work-related burnout complaints. The screening interview was followed by an intake interview, in which a semistructured diagnostic interview was administered face-to-face by a clinical psychologist and the patient completed the Beck Depression Inventory (BDI) [39]. During the semistructured interview the complaint history was assessed and the Composite International Diagnostic Interview (CIDI) [40] was administered. Inclusion criteria were (1) endorsement of the symptoms of neurasthenia, that is, continuous mental and/or physical fatigue and increased fatigability, and at least two other stress complaints out of the following: dizziness, dyspepsia, muscular aches or pains, tension headaches, inability to relax, irritability, and sleep disturbance; (2) a primary role of (a) work-related stressor(s) in the development of complaints as judged by the patient, the referring clinician, and the clinical psychologist; and (3) presence of impaired daily functioning as indicated by (partial) sickness absence which had lasted at least two weeks but less than six months. Exclusion criteria were (1) a primary diagnosis of major depression, social phobia, panic disorder, somatoform disorder other than undifferentiated, posttraumatic stress disorder, obsessive-compulsive disorder, hypomania, or psychotic disorders, all as assessed with the CIDI [40]; (2) severe depressive complaints (i.e., conservatively defined as ≥25 on the BDI); (3) a traumatic event in the past six months; and (4) a medical condition that could better account for the fatigue (e.g., diabetes); (5) excessive alcohol and/or drug use; and (6) pregnancy. The current physiological study was a part of a comprehensive project about psychological and physiological aspects of work-related stress (e.g., de Vente et al. [38]). For this study, patients were refunded for their travel expenses and received a printed report of their baseline blood pressure and heart rate.

As a reference group, forty healthy individuals were recruited by flyers in public places (e.g., libraries and supermarkets; *n* = 29) and among part-time working psychology students (*n* = 11). They were screened by telephone. Participants in good physical health and working for at least 16 hours a week were included in the study. Exclusion criteria were (1) psychiatric illness as determined by the CIDI [40]; (2) currently taking sick leave; (3) a traumatic event in the past six months; (4) a history of immune, diabetic, or other medical disease causing fatigue; (5) excessive alcohol and/or drug use; and (6) pregnancy. Healthy participants were paid 15 euro and received a printed report of their baseline blood pressure and heart rate values after attending the laboratory session and completion of the questionnaires.

### 2.2. Materials

#### 2.2.1. Acute Psychosocial Stressor

**Figure 1:**
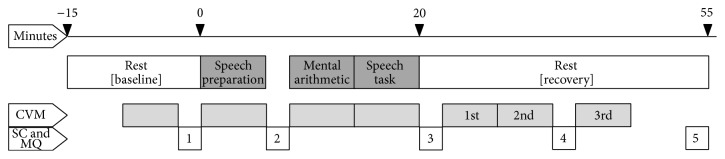
Time diagram of the psychosocial stress procedure. Note: CVM: cardiovascular measurements and SC and MQ: saliva collection for endocrine measures and mood questionnaire.

To study physiological reactivity and recovery, participants were exposed to an acute psychosocial stressor consisting of a speech preparation task, a mental arithmetic task, and a speech task (see Figure 1 for the complete procedure). The speech preparation task consisted of preparing a story about a dramatic social situation in which the participant was unfairly accused of causing damage to the property of others. The mental arithmetic task entailed continuous attention-demanding addition, subtraction, multiplication, and division. For the speech task, the prepared story had to be told in front of the camera and the participant was told that the tape would be analysed. Psychosocial stress procedures have been demonstrated to enhance perceived stress and result in cardiovascular and neuroendocrine reactions (e.g., [30, 41–44]).

#### 2.2.2. Cardiovascular Assessment

Heart rate (HR) and blood pressure (BP) were measured by continuous measurement of finger BP using a Finapres (Ohmeda Finapres type 2300^E^, Blood Pressure Monitor) and the software Vsrrp98 [45]. Systolic blood pressure (SBP), diastolic blood pressure (DBP), HR, cardiac output (CO), and total peripheral resistance (TPR) were calculated using the software Beatscope (version 1.1 [46]). Heart rate variability was calculated using the root mean square of successive differences (RMSSD) of interbeat intervals (IBIs): *√*(1/*n* Σ(IBI_*i*_  −  IBI_*i*−1_)^2^), reflecting mainly high frequency power, and is therefore an adequate measure of the cardiac vagal tone [32, 33]. IBIs were defined as the number of milliseconds between peaks of subsequent systoles in the photoplethysmographic signal, analysed with Vsrrp98 (version 5.4b). The RMSSD based on the IBIs determined in the photoplethysmographic signal was called estimated heart rate variability (EHRV). EHRV based on the photoplethysmographic signal appears to be a valid measure of HRV [47]. Before analyses, the photoplethysmographic signal was inspected visually and artefacts (e.g., movement) and ectopic beats were removed. Mean values of cardiovascular measures were calculated per five minutes. Mean values during the last five minutes of the prestressor baseline phase (see Figure 1) were used as baseline values, indicative of basal functioning. For reactivity, mean values during the stress-inducing tasks were related to the prestressor baseline. For recovery, mean values of the first to third recovery phase (i.e., 6–10 min., 11–15 min., and 21–25 min. poststressor) were also related to the prestressor baseline.

#### 2.2.3. Neuroendocrine Measures and Protocols

Alpha-amylase and cortisol were determined in saliva collected as described by Navazesh [48]. Accordingly, participants refrained from swallowing for a period of four minutes, allowing the saliva to accumulate in the floor of the mouth. The saliva is spitted out into a cup every 60 s. The collection starts with the instruction to void the mouth of saliva by swallowing. Fifteen minutes before the first saliva collection, participants rinse their mouths with water.

Saliva samples were stored on ice until the end of the experiment. Immediately after the session, that is, within 90 min. after collection, saliva was homogenised using a vortex mixer and clarified by centrifugation (10,000 ×g. for 4 min). Aliquots (0.5 mL) with clear supernatant were stored at −20°C until analysis.

Alpha-amylase activity was assayed photometrically (Roche, Almere, Netherlands) after 500-fold dilution using 5 ethylidene-G_7_PNP as substrate. The lower detection limit for amylase was 3 U/L. The amount of free cortisol was determined using enzyme-immunoassay (EIA). Kits were purchased from Diagnostic System Laboratories (DSL, Veghel, Netherlands). The sensitivity of cortisol assay was 1 ng/mL. All samples were assayed in duplo. Intra-assay variability of alpha-amylase and cortisol was 0.4%–2% and 2%–10%, respectively.

Means of the first (−4 min. in relation to the start of the stressor) and second (+5 min.) saliva samples were used as resting values, indicative of basal functioning (see Figure 1). For alpha-amylase, the third (+20 min., i.e., immediately after cessation of the stressor) saliva sample indicated reactivity and the fourth (+35 min.) and fifth (+50 min.) saliva samples recovery. For cortisol, the third (+20 min.) and fourth (+35 min.) saliva samples indicated reactivity, and the fifth (+50 min.) indicated sample recovery.

#### 2.2.4. Psychological Measures and Background Variables

Burnout complaints were measured with the Maslach Burnout Inventory-General Survey (MBI-GS) [49], which consists of three subscales: emotional exhaustion (5 items), depersonalisation (4 items), and professional competence (6 items). Items were scored on 7-point Likert scales (0  =  never to 6 = always/daily) and mean subscale scores were calculated. Higher scores reflect higher levels of emotional exhaustion, distant/cynical attitudes towards work, and professional competence. Cronbach's alpha was 0.85 for emotional exhaustion, 0.81 for depersonalisation, and 0.75 for professional competence in the patient sample. For healthy participants, Cronbach's alpha was 0.83, 0.73, and 0.68, respectively.

Distress complaints were defined as fatigue, depression, anxiety, and stress complaints. Fatigue was measured with the Checklist Individual Strength (CIS) [50]. The CIS consisted of 20, which were scored on a 7-point Likert scale (1 = false to 7 = true). Lower scores indicate lower levels of fatigue. Cronbach's alpha in the current sample was 0.90 in both the patient group and healthy group. Depression, anxiety, and stress were measured with the Depression, Anxiety, and Stress Scales (DASS) [51]. The DASS comprises three subscales of 14 items each, referring to depressive, anxiety, and stress complaints. Severity of complaints during the past week is rated on 4-point Likert scales (0 = not at all/never applicable to 3 = very much/most of the time applicable). Higher scores represent higher levels of complaints. Cronbach's alpha was 0.93 for depression, 0.80 for anxiety, and 0.92 for stress in the patient sample, and 0.83 for depression, 0.72 for anxiety, and 0.94 for stress in the healthy sample.

Mood during the psychosocial stress procedure was measured by the Profile of Mood Scale (POMS) [52]. The Tension (6 items) and Anger (7 items) subscales were selected to measure aspects of negative affect, indicative of subjective stress induction. Items were scored on a five-point scale (0  =  not at all to 4 = very much). Higher scores on the Tension and Anger subscales are indicative of more tension and anger, respectively. Cronbach's alpha at the first administration during the psychosocial stress procedure (i.e., MQ1; see Figure 1) was 0.86 for Anger and 0.82 for Tension in patients, and 0.68 for Anger and 0.74 for Tension in healthy participants. Mood questionnaires were administered five times along with saliva collections (see Figure 1). These mood-dimensions have been previously used to measure the subjective response during stress-inducing tasks [30, 44] and were used as a manipulation check in the present study.

The relevant control variables for cardiovascular and neuroendocrine parameters such as smoking, hours of sleep, and body mass index (BMI) were assessed by questionnaire. Women were also asked to report on menstrual phase, the use of oral contraceptives, and pre-/postmenopausal status.

### 2.3. Procedure

The ethics committee of the Department of Psychology, University of Amsterdam, approved the research protocol and all participants gave written informed consent. Questionnaires regarding biographical information and burnout and distress complaints were completed at home during the week before the psychosocial stress procedure in the laboratory. To control for time of the day effects, all psychosocial stress procedures took place between 14.00 and 16.30 hr. Participants were asked to refrain from eating, smoking, and coffee and tea consumption for at least one hour before the start of the experiment. The blood pressure cuff was attached to the nondominant arm and the arm remained at approximately heart level throughout the session. To prevent pulse dampening, the Finapres was switched off for three minutes during the first and fourth saliva collection. During the whole experimental session, participants remained seated. The questionnaire regarding control variables such as smoking was completed at the start of the session.

### 2.4. Statistical Analyses

To test between-group differences in resting values indicative of basal levels, between-group differences in baseline values of cardiovascular and neuroendocrine measures were assessed by analyses of variance (ANOVA), using a one between-subject (group) factor design.

To test between-group differences in reactivity-recovery and in overall mean activity during the psychosocial stress procedure, mood, cardiovascular, and neuroendocrine reactivity and recovery during the psychosocial stress procedure were examined with ANOVA for repeated measures, using a one within- (time), one between- (group) subject factor design. When the assumption of sphericity was violated, Greenhouse-Geisser-corrected results were reported, resulting in somewhat more conservative testing. When time-group interactions were statistically significant, simple contrasts were employed to explore differences in reactivity and recovery as compared to baseline.

As gender differences in cardiovascular, cortisol, and alpha-amylase activity have been reported previously (e.g., see Kajantie and Phillips [27], for a review; see [53–55]), effect modification of gender was investigated. When effect modification was found, stratified results were presented. As age and BMI are known to be related to physiological outcomes, they were added as covariates to all analyses concerning cardiovascular and neuroendocrine outcomes. In addition, menstrual phase, oral contraceptive use, and menopausal status are known to be related to cortisol [27, 28] and were therefore added as covariates when analysing cortisol in women.

Because of positively skewed data for Anger, EHRV, and alpha-amylase, square root transformed data were analysed, resulting in approximately normal distributions of the data, and skewness and kurtosis were <|2|, except for the kurtosis of Anger which was <3. Two-sided test was performed, applying a significance level of 0.05. All analyses were carried out using SPSS 20. For (square rooted) cardiovascular and (square rooted) neuroendocrine measures, outliers (i.e., values ± >3 SDs of the mean) were removed; the number of outliers was <5%. Some physiological data were missing due to equipment problems (CO, TPR, EHRV; <1%) or insufficient saliva (alpha-amylase, cortisol; 3%–8.5%).

## 3. Results

### 3.1. Sample Characteristics

Sample characteristics of the patient and healthy group are presented in Table 1. Groups differed on gender distribution, *χ* ^2^(1, *n* = 95) = 4.42, *p* = 0.035. Furthermore, male patients were somewhat lower educated, *t*(49) = −3.06, *p* = 0.004, had somewhat higher BMI, *t*(49) = 2.45, *p* = 0.018, and were 7.1 hours more employed, *t*(16.2) = 2.80, *p* = 0.013, than healthy males. Education did not appear to be a confounder in the analyses of group differences in neuroendocrine and immune measures. Hence, the presented results were not adjusted for education. Mean duration of sickness absence in patients was 8.58 (SD = 7.39) weeks. None of the healthy participants was on sickness leave. Three patients (5%) that were using beta-blocker antihypertensive medication were excluded from further analyses. Two patients (4%) used antidepressive medication and four patients (7%) used an anxiolyticum. Five female patients (24%) were using oral contraceptives. Healthy participants, except for eight women (33%) who used oral contraceptives, were medication-free. Three women in the patient group (14%) reported to be in the menstrual phase (days 1–6), three (14%) in the follicular phase (days 7–14), and 10 (48%) in the luteal phase (days 15–28). In the healthy group (missing: *n* = 1), the numbers were eight (33%), four (17%), and seven (29%), respectively. Five patients (24%) and four healthy women (17%) reported having passed their menopause. No statistically significant differences were found in menstrual phase distribution or pre-postmenopausal distribution.

Patients had significantly higher mean scores on all complaints than healthy participants; effect sizes (i.e., Cohen's *d*) were between 1.20 and 2.73; all *p* values < 0.001. An exception was noted for professional competence, for which the between-group effect was small (Cohen's *d* = 0.10) and not statistically significant. All patients scored above the validated cut-off score for the emotional exhaustion subscale of the MBI-GS (i.e., >2.20, [49]), indicating severe exhaustion, and/or the validated cut-off score for CIS (i.e., >76, [56]), indicating severe fatigue. Male and female* patients* did not differ significantly on any of the complaints (all Cohen's *d*'s < 0.50; all *p* values > 0.10).

### 3.2. Mood during the Psychosocial Stress Procedure

**Figure 2:**
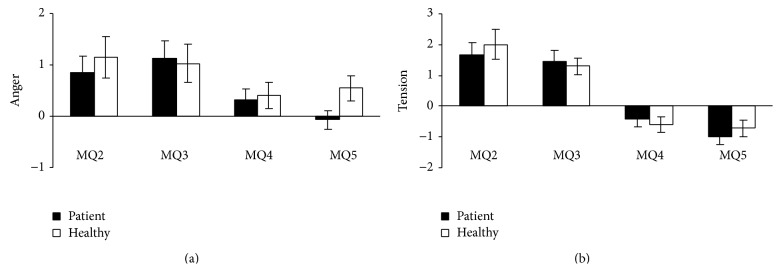
Mean change scores and standard errors of negative affect during the psychosocial stress procedure using the score on MQ1, that is, the mood questionnaire administered during rest, as a reference.

Descriptive information on the subjective stress response during the psychosocial stress procedure is provided in Figures 2(a)-2(b). Anger and Tension changed over time (*F*-values > 10, *p* values < 0.001); they increased during the stressors (simple contrasts Anger: MQ2 versus MQ1: *p* < 0.001, and MQ3 versus MQ1: *p* < 0.001; Tension MQ2 versus MQ1: *p* < 0.001, and MQ3 versus MQ1: *p* < 0.001), supporting subjective stress-induction by the acute psychosocial stress tasks.

### 3.3. Sympathetic, Parasympathetic, and Neuroendocrine Activity, Reactivity, and Recovery

**Figure 3:**
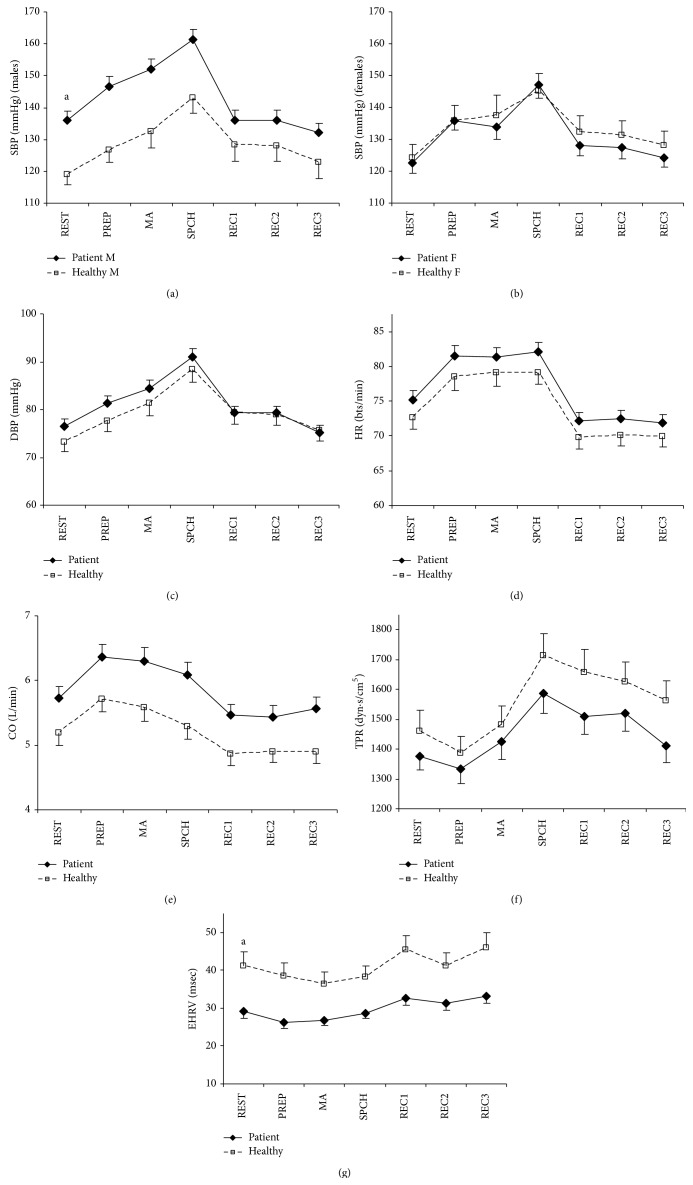
Means and standard errors of cardiovascular measures during the psychosocial stress procedure. Note: SBP: systolic blood pressure; DBP: diastolic blood pressure; HR: heart rate; CO: cardiac output; TPR: total peripheral resistance; EHRV: estimated heart rate variability; REST: baseline rest phase; PREP: speech preparation; MA: mental arithmetic; SPCH: speech task; REC: recovery phase; M: males; F: females. For EHRV the group difference remained statistically significant (*p* values < 0.05) throughout the experiment with no interaction effect of group by time. The group difference during the experiment in CO was marginally significant (*p* = 0.054), with no interaction effect of group by time. ^a^ *p* < 0.05 for group differences in prestressor levels (REST); see also Table 2, results in bold.

**Figure 4:**
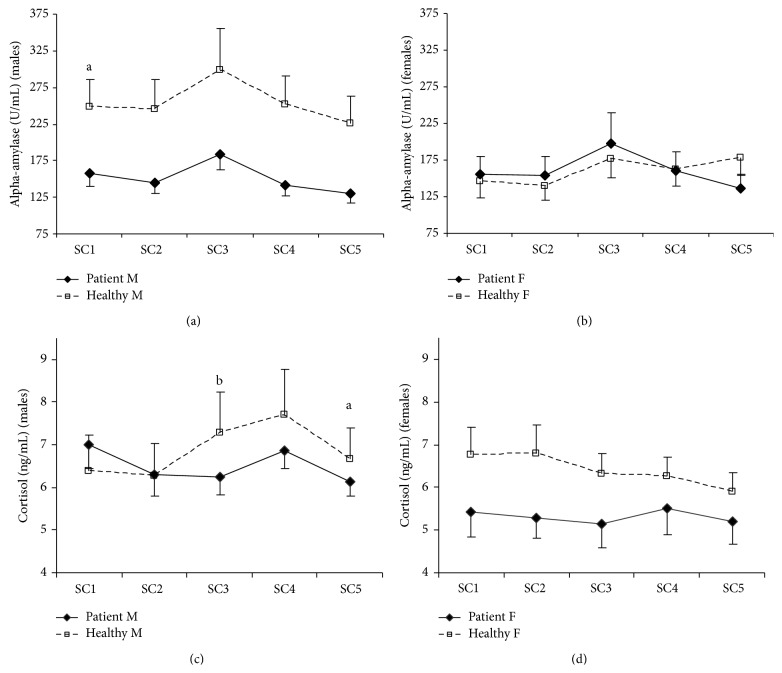
Means and standard errors of neuroendocrine measures during the psychosocial stress procedure. Note: SC: saliva collection; M: males; F: females. To convert salivary cortisol (ng/mL) to System International Units (nmol/L), multiply by 2.76. For alpha-amylase (males), the group difference remained statistically significant (*p* values < 0.05) throughout the experiment with no interaction effect of group by time. ^a^ *p* < 0.05 for group differences in alpha-amylase prestressor levels (REST) or cortisol recovery (SC5; see also Table 2, results in bold). ^b^ *p* < 0.01, for group differences in cortisol reactivity (SC3; see also Table 2, result in bold).

Figures 3(a)–3(g) show means and standard errors of cardiovascular variables, and Figures 4(a)–4(d) demonstrate means and standard errors of neuroendocrine variables during the psychosocial stress procedure. Consistent with the literature, effect modification of gender was found for SBP, alpha-amylase, and cortisol, for which stratified results are presented.

In Table 2, test results of baseline values and reactivity and recovery during the psychosocial stress procedure relative to baseline values are listed. At baseline, patients demonstrated higher SBP (males only), lower EHRV, and lower alpha-amylase (males only) than healthy individuals. In addition, a trend was found for lower cortisol in female patients as compared to healthy females.

All physiological measures changed over time during the psychosocial stress procedure (*F*-values > 3.40,* p* values < 0.05). The observed patterns were consistent with expected activation and recovery due to stress induction. An exception was the observed pattern in cortisol in healthy females, which demonstrated a reduction instead of an increase after baseline. Similar to differences in baseline values, group differences of mean values during the complete psychosocial stress procedure were found for SBP in males, EHRV, and alpha-amylase for males. A trend was found for CO, suggesting higher CO in patients. Since significant time × group interaction effects were absent, these main effects of group support differences in basal activation, independent of acute stress-induction.

Differences in SBP-dynamics and the trends for DBP-dynamics and alpha-amylase-dynamics (females) during the psychosocial stress procedure could not be attributed to either reactivity or recovery (i.e., none of the simple contrasts was statistically significant). For cortisol, healthy males showed earlier and stronger cortisol reactivity immediately after cessation of the stressor (+20 min.; *p* = 0.008) than male patients. Mean reactivity immediately after the stressor of healthy males was 0.91 ng/mL (2.51 nmol/L), which is almost equal to the operational guideline for cortisol reactivity of 1 ng/mL (2.76 nmol/L) [57]. A cortisol reaction could not be observed in male patients at this moment. A trend was found for a similar pattern at the fourth measurement (+35 min.; *p* = 0.080). Mean cortisol reactivity for healthy males at this point was 1.18 ng/mL (3.26 nmol/L), which clearly indicates a cortisol secretory response. Mean cortisol reactivity for male patients was 0.42 ng/mL (1.16 nmol/L), which does not cross the secretory threshold. In addition, at the fifth measurement (+50 min.), cortisol in healthy males had not returned to the baseline level, in contrast to cortisol in male patients (*p* = 0.039). The trend for different cortisol-dynamics (females) during the psychosocial stress procedure could not be attributed to either reactivity or recovery (i.e., none of the simple contrasts was statistically significant).

## 4. Discussion

This study assessed whether burnout is characterised by dysregulation of the sympathetic vagal balance and the HPA axis that may explain the association between burnout and CVD, taking into account gender differences in these mechanisms. Support for predominance of the sympathetic system in burnout was obtained as indicated by elevated basal systolic blood pressure (males only), reduced basal heart rate variability, and a trend for elevated cardiac output in the burnout group as compared to the healthy reference group. The reduction in basal alpha-amylase in male patients is apparently inconsistent with the predicted sympathetic predominance. The latter result is discussed in further detail below. In contrast to prediction, reduced cortisol reactivity to an acute psychosocial stressor was observed in male patients, which suggests hyporeactivity of the HPA axis, rather than hyperactivity. Although the simultaneous predominance of the sympathetic system and hyporeactivity of the HPA axis was not predicted, this pattern has been found previously in the context of chronic stress, burnout, and vital exhaustion and gives rise to unfavourable alterations in immune functioning fostering risk for CVD [4].

Our study further highlights gender differences in cardiovascular functioning and in cortisol reactivity to a psychosocial stressor and in basal alpha-amylase in the context of burnout. Given nonsignificant gender differences in levels of complaints, males seem to develop a more adverse physiological profile as compared to females when they experience work-related stress. More specifically, in the adult life phase roughly between 25 and 60 years, which for females largely covers the premenopausal phase, enhanced cardiac sympathetic activation and hyporeactivity of the HPA-axis are more evident among males than among females with burnout. These gender-specific profiles associated with burnout in this life phase suggest that different mechanisms are at work in men and women with regard to cardiovascular risks. More prominent adverse physiological profiles in men are in line with the findings of Belkic et al. [5], who showed more consistent associations between job strain and CVD in men than in women. Future research may enlighten whether the same adverse profile also emerges in postmenopausal females.

The unexpected finding of reduced basal alpha-amylase-level in male patients deserves further discussion, as it seems to be inconsistent with the other indicators of a basal sympathetic predominance in the male patient group. One may argue that alpha-amylase values were confounded by salivary flow rate. To rule out this possibility of confounding, we tested whether flow rate differed between groups and we reanalysed the data by adjusting for flow rate. Indeed, in line with other studies (e.g., Bosch et al. [58]; Rohleder et al. [59]), no support for confounding was obtained as no differences in flow rate between healthy males and male patients were observed (data not shown), and similar outcomes were obtained when analyses were adjusted for flow-rate (results not shown). A more plausible explanation is that negative affect may have affected basal alpha-amylase levels, as negative associations have been found between resting alpha-amylase and negative affect as well as with avoidance behaviour (e.g., Fortunato et al. [60]). Hence, the group difference in alpha-amylase that we observed may reflect a group difference in a potentially disease-state related basal affective state. It is also possible that, in a resting condition, alpha-amylase reflects a different balance between sympathetic and parasympathetic activation than during a stress reaction. Alpha-amylase in resting conditions may be more strongly influenced by parasympathetic activation than by sympathetic activation. Indeed, animal research has demonstrated that alpha-amylase also increases under parasympathetic activation (see Nater et al. [61], for an overview), which makes sense when one considers that alpha-amylase was first identified as a digestive stimulating hormone (e.g., Ramasubbu et al. [62]). Hence, the higher alpha-amylase level during rest in healthy males may be a reflection of their higher basal parasympathetic activation. This reasoning then sustains that the observation of simultaneous elevated basal cardiovascular activity, reduced basal heart rate variability, and reduced basal alpha-amylase in patients is a result of reduced parasympathetic activity. The stressor, in its turn then, evokes an additional sympathetic response that results in a similar alpha-amylase reaction in both groups. Whether reduced alpha-amylase in a resting condition should be interpreted more as an indication of reduced parasympathetic activation rather than reduced sympathetic activation remains to be elucidated in future research. It is interesting to note, though, that chronic stress in children has also been associated with reduced alpha-amylase in rest [63].

If a relation between burnout and adverse physiological profiles is replicated, these results give rise to several clinical implications. First, in order to keep employees healthy, general awareness of adverse consequences of prolonged job stress and signs of burnout may be increased by educational interventions in the workplace, for both employers and employees. These types of interventions may also focus on solutions to reduce stress. Second, monitoring job stress and other life stress may be added to regular physical health checks in organizations. Our results provide an initial suggestion to carefully monitor signs of (pre)hypertension particularly in male patients reporting work-related stress, as we found elevated SBP among males and a tendency for elevated CO in the group as a whole. Moreover, DBP was 80 mmHg in male patients (results not shown), which is nowadays considered “prehypertension” [64]. Before providing actual guidelines regarding preventive monitoring, further information has to be obtained about the chronicity of these adverse profiles. If changes turn out to be lasting, or worse progressive, early intervention is required, as hypertension poses a main risk factor for cardiovascular disease and mortality (e.g., Yusuf et al. [65]). Third, in case of sickness absence due to burnout, coworkers may be encouraged to keep in touch and support the sick colleague, since coworker support is suggested to promote reduction of burnout complaints [66]. Furthermore, a lack of social support, that is, social isolation, has been associated with more adverse physiological profiles [67] and enhanced risk of CVD [68].

Treatment implications for reduced cortisol responsiveness (in contrast to reduced basal values) are not available. In particular with respect to burnout, insufficient information about the defects in cortisol responsiveness and their potential spontaneous normalisation is available. However, there is initial evidence that psychotherapy results in increases of cortisol in burnout patients [69] and in the stress-related condition PTSD [70]. Future studies may investigate whether psychotherapy or pharmacotherapy such as a low dose of hydrocortisone normalizes cortisol* responsiveness* in burnout as well.

Some methodological issues and limitations of this study deserve consideration. Firstly, we did not recruit our patient sample and healthy sample in a similar way and did not match our samples on background variables. However, to compensate for suboptimal matching, we adjusted the analyses for relevant covariates such as age, gender, body mass index, menstrual phase, oral contraceptive use, and menopausal status, thus ruling out biased outcomes due to sample differences on these variables. Secondly, since the majority of the patient sample consisted of employees working in small- to medium-size enterprises, the reported levels of burnout complaints cannot be generalized to the total Dutch population. Thirdly, despite the fact that we found no statistically significant gender differences on complaints in the patient sample, the male patients seemed to overall report somewhat higher levels of general distress complaints, such as depressive complaints. Consequently, it cannot be ruled out that the gender differences in physiological profiles reflect this tendency for higher general distress complaints in males, rather than reflecting essentially different physiological mechanisms in males and females (e.g., those that are generally linked to hormonal differences [27]). Fourthly, as discussed above, the stress response of healthy females was below the criterion for HPA axis activation, which may have hindered detection of deviant values in women with burnout. Finally, we studied the acute physiological stress response in a laboratory setting, which may not necessarily generalize to a real life work-setting. In support of external validity of our manipulation of the acute physiological stress response is the fact that we used tasks that resemble daily challenges at work. While separate central neuroendocrine circuits have been suggested for different stressors (e.g., Pacák and Palkovits, [71]), the most important distinctions in physiological stress responses are considered to occur between physical and psychological stressors [72–74], supporting the external validity of our acute stress manipulation and its relevance for studying physiological adaptations associated with burnout.

Future research may focus on gender differences in the longitudinal course of sympathetic and parasympathetic adaptation and HPA axis adaptation associated with burnout. Furthermore, longitudinal studies may also focus on explaining inconsistent findings with regard to cardiovascular and neuroendocrine changes associated with burnout. Factors like complaints severity and duration and presence of absence of the stressor seem to be relevant in this respect [18, 21, 37, 72]. Third, future research may aim to clarify the adverse physiological profiles associated with specific aspects of burnout (see, e.g., Marchand et al. [75]) or general distress complaints, such as depressive complaints, that often cooccur with burnout.

In summary, this study indicates that a clinical level of burnout is associated with adverse physiological changes that most likely increase the risk for CVD. More specifically, we found reduced parasympathetic activity and a tendency for elevated cardiac output, which points towards predominance of sympathetic activity in the sympathetic vagal balance. As basal systolic blood pressure was also elevated in males, further support was found for a sympathetic predominance specifically in males. In addition, in males, evidence for hyporeactivity of the HPA axis was found. Consequently, the present study provides support for gender-specific cardiovascular and neuroendocrine profiles associated with burnout. Further longitudinal research is needed in order to assess gender differences in the developmental trajectory of sympathetic and parasympathetic changes and HPA axis hypoactivity associated with burnout and the associations with cardiovascular diseases.

## Figures and Tables

**Table 1:** Characteristics of patients and healthy participants listed for males and females separately [M (SD)/frequency (%)].

	Males		Females	
	Patient (*n* = 34)	Healthy (*n* = 16)	Patient (*n* = 21)	Healthy (*n* = 24)
Age (years)	42.79 (9.70)	38.00 (9.44)	37.95 (9.13)	37.42 (10.09)
Education (1 = primary school, 6 = university)	3.06 (1.39)^a^	4.25 (1.18)^a^	4.29 (1.45)	4.08 (1.38)
Employment (hrs/wk)	38.71 (2.98)^a^	31.63 (9.89)^a^	32.52 (5.75)	28.00 (8.09)
Smoker (yes/no)	7/27 (21/79)	4/12 (25/75)	4/17 (19/81)	4/18 (18/82)
Sleep duration (hours)	7.59 (1.12)	7.93 (0.47)	7.35 (1.58)	7.87 (1.47)
Body mass index (kg/m^2^)	25.92 (3.27)^a^	23.55 (3.17)^a^	23.79 (5.09)	23.19 (2.74)
Emotional exhaustion (MBI-GS, range: 0–6)^b^	4.44 (1.17)	1.25 (0.84)	4.09 (1.43)	1.32 (0.82)
Depersonalisation (MBI-GS, range: 0–6)^b^	2.82 (1.56)	1.23 (0.77)	3.21 (1.28)	1.57 (1.02)
Professional competence (MBI-GS, range: 0–6)	3.81 (1.09)	3.98 (0.89)	3.67 (0.92)	3.76 (0.92)
Fatigue (CIS, range: 20–140)^b^	111.00 (16.79)	43.19 (17.41)	98.83 (19.66)	54.75 (20.75)
Anxiety (DASS, range: 0–42)^b^	8.04 (6.34)	2.19 (2.40)	7.05 (4.06)	2.63 (3.10)
Depression (DASS, range: 0–42)^b^	15.03 (8.31)	3.19 (3.29)	11.21 (6.69)	4.46 (3.78)
Stress (DASS, range: 0–42)^b^	20.40 (9.16)	4.75 (4.36)	17.56 (6.70)	8.71 (8.57)

Note: MBI-GS: Maslach Burnout Inventory—General Survey; CIS: Checklist Individual Strength; DASS: Depression, Anxiety, and Stress Scales. ^a^ *p* < 0.05; ^b^between-group differences stratified by gender: *p* < 0.001.

**Table 2:** Test results comparing prestressor resting values (ANOVA), mean values during the psychosocial stress procedure, and reactivity and recovery (ANOVA for repeated measures) between the patient and healthy group.

	Resting^a^ (group)			Mean during session^a^ (group)			Reactivity and recovery^a^ (group × phase)		
	df's	*F*	*p*	df's	*F*	*p*	df's	*F*	*p*
SBP									
M	1,46	6.57	**0.014**	1,46	4.87	**0.032**	3.3, 153.6	3.11	**0.024**
F	1,41	0.18	0.676	1,41	0.23	0.636	3.7, 151.4	0.68	0.596
DBP	1,90	0.44	0.507	1,90	1.13	0.291	3.5, 310.6	2.08	0.094
HR	1,90	0.97	0.328	1,90	1.39	0.242	3.0, 268.8	0.44	0.723
CO	1,89	1.71	0.195	1,89	3.81	0.054	3.7, 327.9	0.61	0.644
TPR	1,89	0.85	0.360	1,89	1.31	0.256	3.4, 298.2	1.16	0.327
EHRV	1,89	4.60	**0.035**	1,89	5.83	**0.018**	3.7, 332.8	0.50	0.726
AA									
M	1,41	7.14	**0.011**	1,41	6.60	**0.014**	2.0, 83.0	0.21	0.813
F	1,39	0.23	0.634	1,39	<0.01	0.998	2.5, 98.8	2.47	0.077
CORT									
M	1,45	1.38	0.246	1,45	<0.01	0.997	2.0, 89.5	3.32	**0.041**
F	1,34	2.92	0.097	1,34	1.22	0.278	2.1, 72.5	2.61	0.077

Note: Group: mean difference between the patient and the healthy group; group *∗* phase: interaction effect of group × phase of the psychosocial stress procedure; SBP: systolic blood pressure; DBP: diastolic blood pressure; HR: heart rate; CO: cardiac output; TPR: total peripheral resistance; EHRV: estimated heart rate variability; AA: alpha-amylase; CORT: cortisol; M: males; F: females. ^a^All analyses were adjusted for the covariates age, BMI, and gender. Cortisol analyses for females were also adjusted for menstrual phase, oral contraceptive use, and menopausal status. Statistically significant differences are presented in bold and are also indicated in Figures 3 and 4, using superscripts.
